# Prevalence of groundnut dry root rot (*Macrophomina phaseolina* (Tassi) Goid.) and its pathogenic variability in Southern India

**DOI:** 10.3389/ffunb.2023.1189043

**Published:** 2023-12-04

**Authors:** Prince Jayasimha Pamala, R. Sarada Jayalakshmi, K. Vemana, G. Mohan Naidu, Rajeev K. Varshney, Hari Kishan Sudini

**Affiliations:** ^1^ International Crops Research Institute for the Semi-Arid Tropics, Patancheru, Telangana, India; ^2^ Acharya N.G. Ranga Agricultural University, Guntur, Andhra Pradesh, India

**Keywords:** disease incidence, dry root rot, groundnut, molecular variability, pathogenic variability

## Abstract

*Macrophomina phaseolina* is the most devastating and emerging threat to groundnut production in India. An increase in average temperature and inconsistent rainfalls resulting from changing climatic conditions are strongly believed to aggravate the disease and cause severe yield losses. The present study aims to conduct a holistic survey to assess the prevalence and incidence of dry root rot of groundnut in major groundnut growing regions of Southern India, *viz*., Andhra Pradesh, Telangana, Karnataka, and Tamil Nadu. Furthermore, the pathogenic variability was determined using different assays such as morphological, cultural, pathogenic, and molecular assays. Results indicate that disease incidence in surveyed locations ranged from 8.06 to 20.61%. Both temperature and rainfall played a major role in increasing the disease incidence. The pathogenic variability of *M. phaseolina* isolates differed significantly, based on the percent disease incidence induced on cultivars of JL-24 groundnut and K-6 groundnut. Morphological variations in terms of growth pattern, culture color, sclerotia number, and sclerotia size were observed. The molecular characterization of *M. phaseolina* isolates done by ITS rDNA region using ITS1 and ITS4 primers yielded approximately 600 bp PCR amplicons, sequenced and deposited in GenBank (NCBI). Molecular variability analysis using SSR primers indicated the genetic variation among the isolates collected from different states. The present investigation revealed significant variations in pathogenic variability among isolates of *M. phaseolina* and these may be considered important in disease management and the development of resistant cultivars against groundnut dry root rot disease.

## Introduction

Groundnut (Peanut; *Arachis hypogaea* L.) is a major oil seed and food legume crop cultivated in tropical and subtropical areas of the world. It is the fourth most important source of edible oil and the third richest source of vegetable protein. It contains 48-50% oil and 26-28% protein and is a rich source of dietary fiber, minerals, and vitamins (Prasad et al., 2009). It is commercially grown between 40°N and 40°S latitude. Globally, the crop is raised on 29.59 million hectares with a total production of 48.75 million MT (FAOSTAT, 2020). In India, groundnut is grown in an area of 4.8 M ha with a production of 9.9 MT and an average productivity of 2.06 tonnes ha^-1^ (Indiastat, 2020). The major groundnut-growing states are Gujarat, Andhra Pradesh, Telangana, Tamil Nadu, Karnataka, Rajasthan, and Maharashtra. These states constitute approximately 80% of India’s area and are responsible for approximately 80% of the country’s groundnut production (Directorate of Economics and Statistics, 2020).

Groundnut production is decreasing gradually because of various biotic and abiotic stresses. Biotic stresses such as fungal, bacterial, and viral diseases play a major role in yield reduction. The soilborne diseases caused by fungal pathogens are very important, and several of them have the potential to cause significant yield losses in groundnut production (Ganesan and Sekar, 2012). The *Macrophomina phaseolina* is an important pathogen that causes dry root disease (Chakrabarty et al., 2005); it is distributed globally, and groundnut crops at all stages are susceptible to infection. This pathogen has a wide host range, and it infects more than 500 economically important crops such as legumes (chickpea, soybean, mungbean, pigeon pea, cowpea, and urdbean (Pandey and Basandrai, 2021)), corn, sorghum, cotton, and groundnut (Gaikwad and Rajurkar, 2018). The fungus is responsible for different disease symptoms, namely, charcoal rot, root rot, seedling blight, foliage blight, dry rot, pod rot, and seed rot, and causes considerable yield losses in crops (Kumar et al., 2020).

Groundnut dry root rot is gaining importance in the changing climatic scenario, especially when the standing crop is exposed to high temperature and moisture stress and this phenomenon looks very similar to chickpea dry root rot (Sharma and Pande, 2013). The disease is becoming more intense in humid tropical areas due to increasing temperatures and more frequent moisture stress. The increasing incidence of dry root rot in various locations over the last years was strongly influenced by rising temperatures (Savary et al., 2011). The most favorable temperatures for infection, colonization, and development of the pathogen ranged between 25 to 35°C (Srinivas et al., 2017). Pande et al. (2010) observed that the incidence of dry root rot disease increased manyfold in the last few years due to continuous and severe moisture stress in semi-arid tropical regions. High day temperatures above 30°C and dry soil conditions at flowering and pod development rapidly increase the severity of dry root rot (Sharma et al., 2016).

Morphological and cultural examinations and molecular techniques were used to characterize *M. phaseolina* isolates from various legume crops (Babu et al., 2007; Sharma et al., 2012). Understanding the disease epidemiology and host-pathogen interactions is greatly dependent on knowledge of the diversity of the pathogen at the crop field level (Prasad et al., 2011). Variations in morphological and cultural characteristics of dry root rot pathogen and differences in the pathogenicity or host preference among isolates have previously been reported (Aghakhani and Dubey, 2009; Sharma et al., 2012; Gowdra et al., 2015; Gade et al., 2018). A recent comprehensive study conducted in India and Myanmar used multiple methods, such as paper towel assay, glasshouse assay, and field conditions, to analyze mungbean dry root rot, incited by *M. phaseolina*. The study indicated cultural and pathogenic variability and further identified three accessions, namely, VI001244AG, VI001509AG, and VI001400AG, with good levels of resistance (Pandey et al., 2021).

Molecular techniques, such as random amplified polymorphic DNA (RAPD) analysis, use of species-specific primers, loop-mediated isothermal amplification (LAMP) based detection, and internal transcribed spacers (ITS) of 18S rRNA (Ghosh et al., 2017), are commonly used to identify *M. phaseolina* (Babu et al., 2010). Genetic diversity of *M. phaseolina* has been detected using simple sequence repeats (SSR) molecular markers in cotton, soybean (Jana et al., 2005), and chickpea (Walunj et al., 2018). Only a few molecular studies have been conducted especially on legumes to assess the genetic diversity in *M. phaseolina* isolates in India using DNA fingerprinting and sequencing techniques (Sharma et al., 2012; Pandey et al., 2021).

Particularly in groundnut, few studies have reported on the occurrence and distribution (Moradia and Khandar, 2011; Rajamohan and Balabaskar, 2012; Muthukumar et al., 2014; Veena et al., 2019) and on the morphological and cultural characterization of pathogens (Gaikwad and Rajurkar, 2018).

Epidemiological studies on dry root rot disease concerning the predisposition of groundnut plants to climate change variables are scarce, including the diversity of pathogenic isolates. Thus, the objectives of this research were to: (1) understand the prevalence and incidence of dry root rot in groundnut growing areas of Southern India, and (2) assess pathogenic, morphological, cultural, and molecular characteristics of *M. phaseolina* isolates from groundnut in four states of Southern India.

## Materials and methods

### Survey on the root rot incidence of groundnut in Southern India

A field survey was conducted during *Kharif* (rainy season: June/July-September/October) 2019 to record the occurrence of dry root rot disease in groundnut growing areas of Southern India, *viz*., Andhra Pradesh, Telangana, Karnataka, and Tamil Nadu. In each state, two districts were included and in each district, 6 to 8 villages were surveyed based on major crop sown particulars. Four 1m^2^ quadrants were randomly selected in each field with the diagonal quadrant transect method, and based on above-ground symptoms, the dry root rot-affected plants were counted in each quadrant. Disease incidence was calculated by counting the infected and the total number of plants in each quadrant (Muthukumar et al., 2014).


$$\text{Percent disease incidence}=\frac{\text{Number of infected plants}}{\text{Total number of plants}}\times 100$$

The relationship between mean percent disease incidence and weather conditions, such as temperature, relative humidity, and rainfall in each state, was analyzed. Weather data was obtained from the nearest meteorological station in the surveyed locations.

### Isolation and identification of dry root rot pathogen

The dry root rot pathogen *M. phaseolina* was isolated from the infected root region of groundnut plants collected during the survey, by tissue segment method using potato dextrose agar (PDA) medium (HiMEDIA). The infected root portion of the plant was excised with a sterilized blade into small bits of 1 cm. The surface was sterilized by dipping in 0.1% HgCl_2_ for 1 min and then washed three times in sterile distilled water (SDW) before plating onto PDA (Rangaswami, 1972). The Petri dishes were incubated at 28 ± 2°C for periodical observations. Microscopic observation was also performed to confirm the isolates as *M. phaseolina* by using an Olympus BX 53 microscope equipped with a digital camera DP 72 (Olympus).

### Mass multiplication of *M. phaseolina*

A total of 60 *M. phaseolina* isolates were isolated (14 or 16 isolates from each state), and the isolates were individually multiplied on sorghum grains (Aghakhani and Dubey, 2009). The sorghum grains were soaked overnight and air-dried at room temperature to remove excess moisture. Then, the grains (250 g) were put into 500 ml conical flasks. The mouth of each flask was plugged with cotton plugs and sterilized twice in an autoclave at 121°C for 15 min at 15 pounds per square inch. After cooling, the actively growing mycelial discs of the *M. phaseolina* isolates were inoculated into each flask separately under a laminar airflow chamber, and the flasks were incubated at room temperature (28 ± 2°C) for 15 days in a BOD incubator. The flasks were shaken on alternate days for uniform colonization of the grains (Iqbal and Mukhtar, 2014). The obtained inoculum was used to determine the pathogenic variability.

### Pathogenic variability of *M. phaseolina* isolates of groundnut collected from Southern India

Pathogenic variability of all the 60 *M. phaseolina* isolates was carried out by soil infestation technique in an earthen pot under controlled environmental conditions (Gade et al., 2018) by using cultivars JL-24 (Phule Pragati) and K-6 (Kadiri-6), with three replications for each isolate in a completely randomized design. Sterilized soil filled in 6-inch diameter earthen pots and inoculum was mixed in soil with each isolate of *M. phaseolina* in 1:9 proportions (inoculum + soil) 10 days before sowing. Groundnut cultivar JL-24 is bold-seeded, has wider adaptability, and early maturity, and K-6 is of the semi-spreading type, has uniform early maturity, and is a high pod-yielded cultivar. Both cultivars are suitable for areas with assured rainfall and irrigation facilities and not suitable for low rainfall areas (Ikisan, 2021). Higher dry root rot disease incidence was observed in several surveyed locations previously on these two varieties such as JL-24 (Muthukumar et al., 2014) and K-6 (Veena et al. 2019). Seeds were surface sterilized with 0.1% HgCl_2_ for 2 min, followed by three serial washings in SDW, and then sown at a rate of five seeds per pot. Pots without inoculum served as controls. Pots were placed in a greenhouse at 28 ± 2°C with regular and uniform watering for optimal susceptibility to disease. The dry root rot incidence was recorded 45 days after sowing, and the percent disease incidence was calculated. Based on the percent disease incidence in the host, the *M. phaseolina* isolates were categorized into four groups: weakly pathogenic, moderately pathogenic, strongly pathogenic, and aggressively pathogenic (Gade et al., 2018).

### Morphological and cultural characteristics of dry root rot pathogen

Morphological and cultural characteristics of all the 60 isolates were studied on Petri dishes with PDA medium under *in vitro* conditions (Chiranjeevi et al., 2021). The mycelial discs of 5 mm diameter made from the margins of actively growing culture were inoculated in the center of 90 mm Petri dishes containing 15 ml of PDA. Each treatment was replicated thrice, and the inoculated dishes were incubated at 28 ± 2°C. All the cultures were observed at 12-hour intervals for monitoring and recording the mycelial growth rate and time of sclerotial initiation (Aghakhani and Dubey, 2009). After 7 days of inoculation, growth pattern, culture color, and presence or absence of aerial mycelium were recorded. Slides were prepared from 7-day-old cultures to record the microscopic observations such as the shape of sclerotia, size of sclerotia (μm) (50 sclerotia were randomly selected), and the number of sclerotia per 10X microscopic field using a Q-capture image analyzer (Sharma et al., 2012).

### Molecular variability of *M. phaseolina* (groundnut dry root rot) isolates collected from Southern India

#### Genomic DNA extraction

The genomic DNA was extracted from the *M. phaseolina* mycelium using the CTAB method with slight modifications (Murray and Thompson, 1980). Briefly, all the 60 isolates were grown in test tubes containing potato dextrose broth (PDB) (HiMEDIA) plugged with cotton plugs and kept for incubation at 28 ± 2°C in a 45° slant position. Then, 5-day-old cultures of dry root rot pathogen were used for DNA extraction. The mycelial mat was removed from the PDB and transferred onto sterile blotting paper to remove excess moisture. The mycelial mat was transferred to pre-chilled mortar and pestle and ground into a fine solution by adding 1 μl of CTAB buffer [1M Tris–HCl (pH 8.0), 5M NaCl, 0.5M ethylenediaminetetraacetic acid (EDTA; pH 8.0) and 2% CTAB]. The solution was transferred into 2 ml microcentrifuge tubes (Eppendorf) and incubated in a water bath at 65°C for 45 min with gentle intermittent shaking by hand for 30 sec. After incubation, 2 μl of RNase A was added, mixed well with vortex for 30 sec, and incubated at 37°C for 15 min. An equal volume of phenol:chloroform:isoamyl alcohol (25:24:1) was added, gently mixed to denature proteins, and centrifuged at 12,857 *g* for 10 min. The supernatant was transferred into new 2 ml centrifuge tubes and an equal volume of chloroform:isoamyl alcohol (24:1) was added, mixed, and centrifuged at 12,857 *g* for 10 min. DNA was precipitated with 0.6 volume of chilled isopropanol and 0.1 volume of 7.5 M ammonium acetate, incubated in ice for 45 min, and centrifuged at 18,514 *g* for 15 min. The pellets were washed twice with chilled 70% ethanol, dried at room temperature, resuspended in 100 μl sterile TE (1M Tris–HCl buffer and 0.5M EDTA; pH 8), and stored at -20°C. Isolated DNA samples were run in 0.8% agarose gel containing ethidium bromide for 45 min at 60 V in 1X TAE buffer to check the quality and quantity of DNA.

### Molecular identification of *M. phaseolina* isolates

PCR-based molecular characterization was carried out by amplifying the rDNA-ITS region of all 60 *M. phaseolina* isolates using universal fungal primers, viz., ITS1 (5'-TCC GTA GGT GAA CCT GCG G-3') and ITS4 (5'-TCCTCCGCTTATTGATATGC-3') (White et al., 1990). For this, a 2X PCR Taq Master mix (Applied Biological Materials, Richmond, Canada) containing dNTPs, DNA polymerase, buffer and MgCl_2_, primers (Integrated DNA Technologies, Coralville, US), and nuclease-free water were used. The PCR thermal cycler (Bio-Rad, California, US) reaction contained 50 ng genomic DNA, 1X PCR Taq Master mix, and 0.25 μM of each primer in a 25 μl reaction volume. The PCR program was as follows: 1 cycle at 94°C for 5 min, 35 cycles at 94°C for 1 min followed by a cycle at 55°C for 1 min and another at 72°C for 2 min, and 1 cycle at 72°C for 5 min, and then the products were held at 4°C. The PCR products were analyzed on 1% agarose gel electrophoresis for 45 min at 60 V in 1X TAE buffer (40mM Tris base, 20mM Acetic acid, and 1mM EDTA), stained with ethidium bromide, and photographed by using Gel Documentation System (Bio-Rad, California, US). PCR amplicons were purified using NucleoSpin Gel and PCR clean-up kit (Macherey-Nagel, Düren, Germany) and sequenced at eurofins Genomics facility.

### Simple sequence repeats analysis

The SSR primers used for *M. phaseolina* (Walunj et al., 2018) were screened against the 60 pathogenic isolates. Five SSR primers were selected and were synthesized from Integrated DNA Technologies, US. Briefly, the reaction volume consisted of a 20 μl mixture containing 1 μl of 50 ng of genomic DNA, 10 μl 2X PCR Taq Master mix, 1 μl of each primer, and 7 μl of nuclease-free water. The PCR was performed as described above, but the annealing temperature set for each primer was 1 min (**Table 1**). The PCR products were electrophoresed on agarose gel (2%) in TAE buffer (1X) at 70 V for one hour, and 1 kb and 100 bp DNA ladders were used as markers. Amplification was done twice before final scoring and the primers, which were reproducible and scorable, were used in the analysis.

**Table 1:** Primer sequences and analysis of polymorphism obtained with simple sequence repeat (SSR) primers in isolates of *M. phaseolina*.

Primer	Sequence (5′-3′)	Annealing temperature (°C)	Total bands (No.)	Polymorphism (%)	Size range of amplicons (kb)
MB 9	F-TGGCTGGGATACTTGTGTAATTG R-TTAGCTTCAGAGCCCTTTGG	52.8	4	100.0	0.2–1.1
MB 10	F-TATCGAGTCCGGCTTCCAGAAC R-TTGCAATTACCTCCGATACCAC	54.0	7	85.7	0.3–1.1
MB 11	F-GTGGACGAACACCTGCATC R-AGATCCTCCACCTGCATC	50.3	9	88.9	0.2–1.2
MB 13	F-GGAGGATGAGCTCGATGAAG R-CTAAGCCTGCTACACCCTCG	50.3	9	77.8	0.2–1.4
MB 17	F-ACTGATTCACCGATCCTTGG R-GCTGGCCTGACTTGTTATCG	48.0	4	100.0	0.2–1.0
		Total	33		
		Mean	6.6	90.7	

### Scoring and data analysis

The reproducible and scorable DNA bands amplified from different SSR primers were used to analyze genetic variability present in the 60 *M. phaseolina* isolates. Data were scored based on the binary character of absence (coded as 0) or presence (coded as 1) of each band for all the isolates in each primer. Dissimilarity matrices were calculated for binary data using the Jaccard coefficient in DARwin software version 6, and cluster analysis was performed to construct the dendrogram by the unweighted neighbor-joining method (Perrier and Jacquemoud-Collet, 2006).

## Results

### Survey and incidence of dry root rot

**Figure 1:**
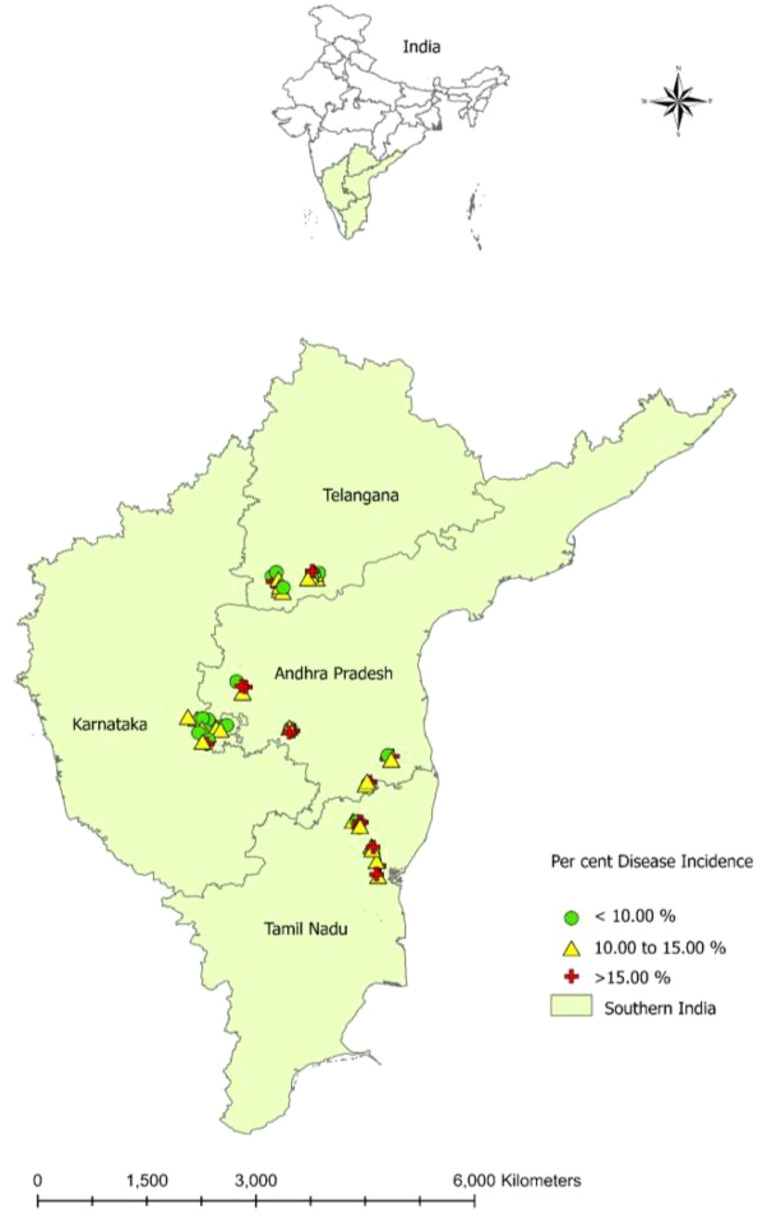
Prevalence of groundnut dry root rot disease in southern India during *Kharif* (rainy season) - 2019.

The dry root rot disease incidence in surveyed areas in southern India ranged from 8.06 to 20.61% (**Table 2**; **Figure 1**). Furthermore, the Tamil Nadu state recorded the highest mean dry root rot incidence of 14.91%, followed by Andhra Pradesh (13.93%) and Karnataka (11.05%). The lowest mean percent disease incidence was recorded in Telangana (10.99%).

**Table 2:** Prevalence of dry root rot disease of groundnut in major crop growing areas of southern India (rainy season/*Kharif* 2019).

State (Percent Mean Disease Incidence)	Districts	Mandal/ Taluk	Village	Farming situation	Soil type	Variety	Isolate number	GPS coordinates		Disease Incidence (%)	Mean Disease Incidence (%)
								Latitude	Longitude		
ANDHRA PRADESH (13.93)	Ananthapuram	Kadiri	ARS	Irrigated	Sandy loam	Kadiri-6	GRb01	14.1114	78.1474	9.73	14.09
			Allugundu	Rainfed	Sandy loam	Kadiri-6	GRb02	14.0677	78.1645	15.68	
			Murthypalli	Rainfed	Sandy loam	Kadiri-6	GRb03	14.1213	78.1434	14.28	
			Kareddypalli	Irrigated	Sandy loam	Kadiri-6	GRb04	14.0589	78.1335	18.74	
		Kudair	Brahmanapalli	Irrigated	Sandy loam	Kadiri-6	GRb05	14.7188	77.4495	13.48	
			Jallipalli	Irrigated	Sandy loam	Kadiri-6	GRb06	14.8095	77.3481	9.28	
			Kommuru	Rainfed	Sandy loam	Kadiri-6	GRb07	14.7276	77.4814	16.23	
			Kuderu	Rainfed	Sandy loam	Kadiri-6	GRb08	14.7424	77.4257	15.32	
	Chittoor	S.R. Puram	Kannikapuram	Irrigated	Clay loam	Narayani	GRb09	13.2972	79.2822	8.91	13.76
			Kothapallimitta	Rainfed	Sandy loam	Narayani	GRb10	13.2893	79.2637	13.95	
			Marripalli	Rainfed	Sandy loam	Narayani	GRb11	13.3039	79.3199	16.12	
			Muchalamarri	Rainfed	Sandy loam	Narayani	GRb12	13.3189	79.2931	12.76	
		Yerpedu	Bandarupalli	Irrigated	Clay loam	TAG 24	GRb13	13.6877	79.6476	16.84	
			Bokkasampalem	Rainfed	Sandy loam	Narayani	GRb14	13.6798	79.6265	17.45	
			Manna samudram	Irrigated	Sandy loam	Narayani	GRb15	13.6977	79.6154	9.82	
			Ramalingapalli	Irrigated	Sandy loam	TAG 24	GRb16	13.6622	79.6444	14.20	
KARNATAKA (11.05)	Chitradurga	Challakare	Gowripura	Rainfed	Sandy loam	GPBD 4	GRb17	14.2409	76.9283	9.67	10.69
			Kydikunta	Rainfed	Sandy loam	GPBD 4	GRb18	14.2006	76.9817	11.90	
			Mahadevapura	Rainfed	Sandy loam	Kadiri-6	GRb19	14.2121	76.9294	14.40	
			Pillahalli	Rainfed	Sandy loam	GPBD 4	GRb20	14.2237	76.9558	9.75	
		Hiriyur	Halagaladdi	Rainfed	Sandy loam	GL24	GRb21	14.0767	76.8907	9.16	
			Karidasarahalli	Rainfed	Sandy loam	GPBD 4	GRb22	14.0678	76.9295	11.53	
			Maddihalli	Rainfed	Sandy loam	GL24	GRb23	14.0972	76.9364	8.40	
	Tumkur	Pavagada	Avasikere	Rainfed	Sandy loam	Kadiri-6	GRb24	14.0828	77.0477	8.33	11.41
			Kadirehalli	Rainfed	Sandy loam	Kadiri-6	GRb25	14.1347	77.0475	13.82	
			Madde	Irrigated	Clay loam	Kadiri-6	GRb26	14.0545	77.0753	8.06	
			Thumakunte	Rainfed	Sandy loam	Kadiri-6	GRb27	14.0601	77.0532	11.01	
		Sira	Bejjihalli	Rainfed	Sandy loam	GPBD 4	GRb28	14.0487	76.9072	9.52	
			Lakkanahalli	Rainfed	Sandy loam	Local	GRb29	14.0312	76.9299	15.63	
			Thimmanahalli	Rainfed	Sandy loam	GPBD 4	GRb30	14.0363	76.9176	13.49	
TAMIL NADU (14.91)	Tiruvannamalai	Arni	Athimalaipattu	Rainfed	Sandy loam	JL 24	GRb31	12.7388	79.1804	13.59	15.93
			Kongarampattu	Irrigated	Clay loam	JL 24	GRb32	12.7526	79.1692	9.68	
			Kilnagar	Irrigated	Sandy loam	Local	GRb33	12.7173	79.2014	17.84	
			Melnagar	Irrigated	Clay loam	VGI 2	GRb34	12.7084	79.1932	15.62	
		Polur	Santhavasal	Rainfed	Sandy loam	JL 24	GRb35	12.6431	79.1652	20.61	
			Sedarampattu	Rainfed	Sandy loam	VGI 2	GRb36	12.6747	79.1826	14.75	
	Villupuram	Gingee	Kanjanur	Rainfed	Sandy loam	VGI 2	GRb37	12.0704	79.4643	18.46	13.90
			Ottampattu	Rainfed	Sandy loam	Local	GRb38	12.1531	79.4274	11.81	
			Tokavadi	Irrigated	Sandy loam	Local	GRb39	11.9313	79.4508	10.83	
			Venkatesapuram	Rainfed	Sandy loam	JL 24	GRb40	11.9436	79.4281	15.15	
		Melmalaiyanur	Kannalam	Irrigated	Clay loam	Local	GRb41	12.3341	79.3838	12.12	
			Mannur	Irrigated	Sandy loam	VGI 2	GRb42	12.2981	79.3729	9.35	
			Sathaputhur	Irrigated	Sandy loam	VGI 2	GRb43	12.3247	79.3644	14.84	
			Valathy	Rainfed	Sandy loam	VGI 2	GRb44	12.3467	79.3784	18.60	
TELANGANA (10.99)	Nagarkurnool	Balmoor	Gattuthummen	Irrigated	Clay loam	Kadiri-6	GRb45	16.4226	78.5708	9.60	11.33
			Godal	Rainfed	Sandy loam	Kadiri-6	GRb46	16.3609	78.5372	11.66	
			Jinkunta	Rainfed	Sandy loam	Local	GRb47	16.4316	78.5138	11.59	
			Thummanpet	Irrigated	Sandy loam	Kadiri-6	GRb48	16.3982	78.4928	9.81	
		Talkapally	Boppally	Irrigated	Clay loam	Kadiri-6	GRb49	16.3484	78.4571	9.44	
			Gattunallykuduru	Rainfed	Sandy loam	Local	GRb50	16.4057	78.4402	10.92	
			Peddur	Irrigated	Sandy loam	Kadiri-6	GRb51	16.3715	78.4391	11.80	
			Kammareddypalli	Irrigated	Sandy loam	Kadiri-6	GRb52	16.4134	78.5069	15.82	
	Wanaparthy	Kothakota	Amadabakula	Irrigated	Sandy loam	Local	GRb53	16.3383	77.9351	9.37	10.66
			Apparaala	Rainfed	Sandy loam	Kadiri-6	GRb54	16.2947	77.8907	15.40	
			Kanimetta	Irrigated	Sandy loam	Kadiri-6	GRb55	16.4351	77.9395	8.66	
			Sankireddypalli	Rainfed	Sandy loam	Kadiri-6	GRb56	16.3492	77.9678	11.16	
		Pebbair	Chelimilla	Rainfed	Sandy loam	Kadiri-6	GRb57	16.1923	77.9931	10.81	
			Kambalapur	Irrigated	Sandy loam	Kadiri-6	GRb58	16.2159	78.0179	10.35	
			Kanchiravupalli	Irrigated	Sandy loam	Kadiri-6	GRb59	16.2286	78.0022	11.20	
			Pebbair	Rainfed	Sandy loam	Local	GRb60	16.2071	78.0431	8.34	

In Andhra Pradesh, dry root rot disease incidence varied from 8.91 to 18.74% (**Table 2**). Among the different locations, Kareddypalli in Ananthapuram district registered the maximum incidence of the disease (18.74%), followed by Bokkasampalem (Chittoor) with 17.45%, and the minimum disease incidence of 8.91% was recorded in Kannikapuram of Chittoor district.

In Karnataka, the incidence of dry root rot varied from location to location (8.06 to 15.63%), and the maximum disease incidence was recorded in Lakkanahalli (15.63%) of Tumkur district, followed by Mahadevapura (14.40%) of Chitradurga district. The minimum root rot incidence was observed in Madde (Tumkur) at 8.06% (**Table 2**).

In Tamil Nadu, the dry root rot disease ranged from 9.35 to 20.61% (**Table 2**). The maximum disease incidence was observed in Santhavasal of Tiruvannamalai district at 20.61%, followed by Valathy (18.60%) of Villupuram, and the minimum dry root rot disease incidence was recorded in Mannur at 9.35% of Villupuram district.

In Telangana, the disease incidence ranged from 8.35 to 15.82%, and the maximum dry root rot incidence was noticed in Kammareddypalli of Nagarkurnool district with 15.82%, followed by Apparala (15.40%) of Wanaparthy district. The minimum root rot incidence was observed in Pebbair of Wanaparthy district at 8.35%(**Table 2**).

**Figure 2:**
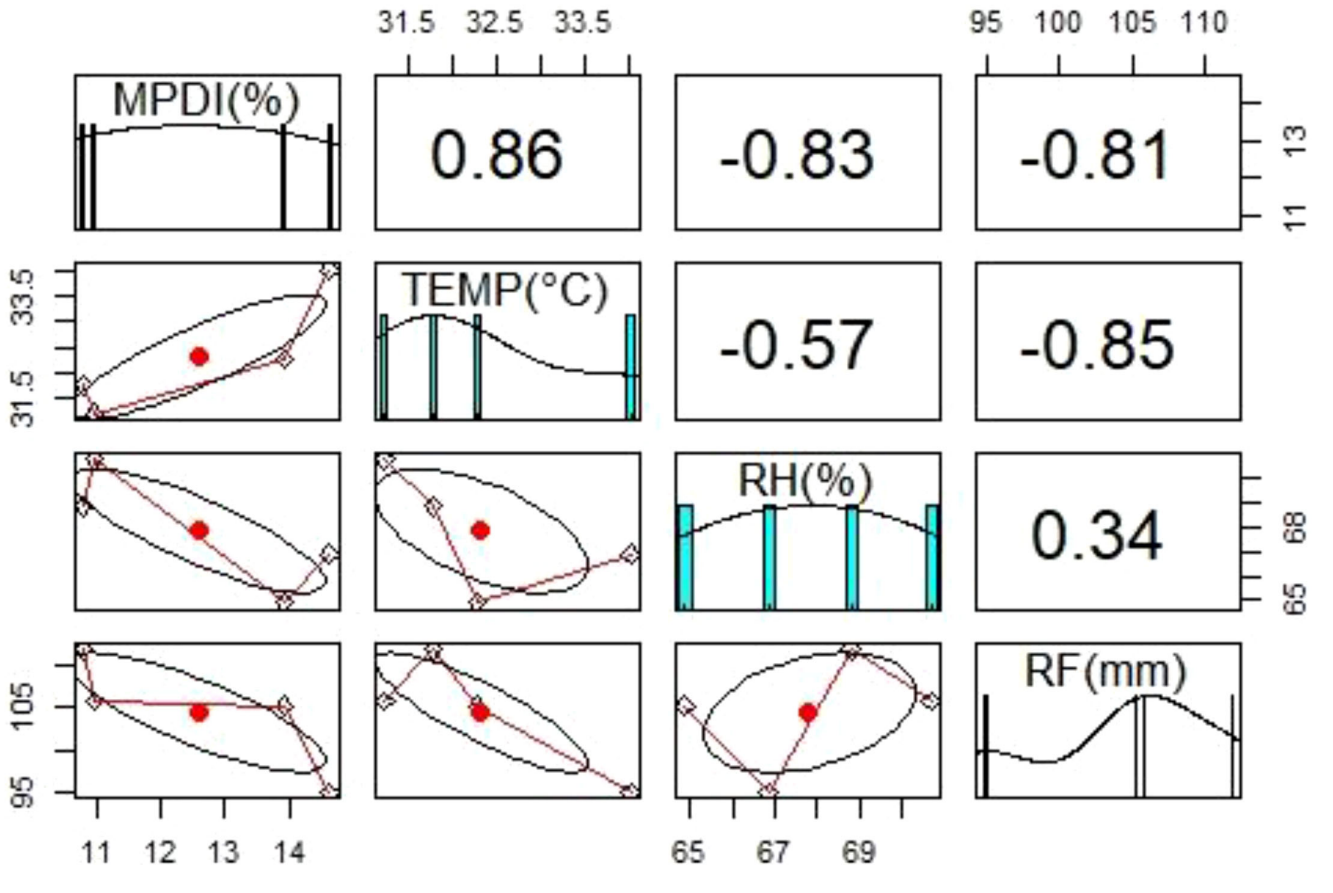
Diagrammatic representation of the relationship between mean percent disease incidence (MPDI) of groundnut dry root rot and weather parameters such as temperature (TEMP), relative humidity (RH), and rainfall (RF) during Kharif (rainy season) 2019 in Southern India.

The differences in disease incidence might be due to variations in weather conditions (temperature, relative humidity, and rainfall) in each state. Significant relationships were found between mean percent disease incidence, mean temperature, mean relative humidity, and mean rainfall (**Figure 2**). There was a positive correlation (+0.86) between mean temperature and mean percent disease incidence: the higher the temperature, the higher the disease incidence. Whereas a negative correlation between mean percent disease incidence with mean relative humidity (-0.83) and mean rainfall (-0.81), low disease incidence was observed with high relative humidity and high rainfall.

### Symptoms of groundnut dry root rot disease

The groundnut dry root rot-infected plants showed typical symptoms such as withering and drying plants. The infected plants, which could be easily pulled out from the soil due to lack of lateral and finer roots, showed blackening of the taproot and shredding of bark. In addition, the bark was coming out in the form of flakes, and the presence of microsclerotia was observed. When split open, the root portion showed blackish discoloration of the vascular system.

### Isolation and identification of dry root rot pathogen

**Figure 3:**
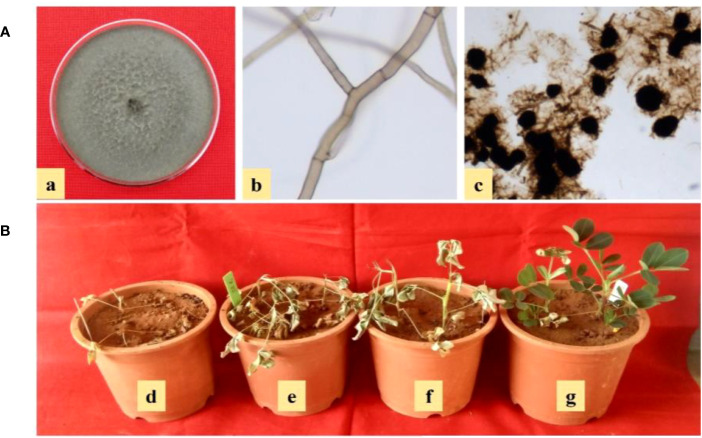
**(A)** Characteristics of *M. phaseolina* on PDA (a) grey to black culture color with aerial mycelium, (b) microscopic characteristics such as hyphal branching at right angle and formation of constriction near the point of branching, and (c) presence of microsclerotia; **(B)** Pathogenic variability of *M. phaseolina* isolates of groundnut collected from Southern India (d-g) aggressively pathogenic (GRb52), strongly pathogenic (GRb11), moderately pathogenic (GRb23), and weakly pathogenic (GRb15).

The pathogen was isolated from diseased plants on PDA plates, and after 2-3 days of incubation, grey to black culture with aerial mycelium was observed, and isolates were purified by hyphal tip technique (Rangaswami, 1972). Microscopic observations such as hyphal branching at the right angle, formation of constriction near the point of branching, and presence of microsclerotia for all the isolates were observed (**Figure 3A**), and the isolates were designated as GRb01 to GRb60 for further studies (**Table 2**).

### Pathogenic variability among *M. phaseolina* isolates of groundnut collected from Southern India

The pathogenic variability of *M. phaseolina* isolates differed significantly, and the 60 isolates were categorized into weakly pathogenic, moderately pathogenic, strongly pathogenic, and aggressively pathogenic groups based on percent dry root rot induced on cultivars of JL- 4 groundnut and K- 6 groundnut (**Table 3**; **Figure 3B**). On cultivar JL-24, the majority (60%) of the isolates were grouped into the moderately pathogenic category, with an average of 21-50% root rot incidence, 15 isolates (GRb-01, GRb-08, GRb-10, GRb-12, GRb-14, GRb-15, GRb-18, GRb-22, GRb-30, GRb-32, GRb-37, GRb-40, GRb-49, GRb-54, and GRb-56) were grouped into the weakly pathogenic category, with an average of 1-20% root rot, and 8 isolates (GRb-11, GRb-16, GRb-20, GRb-27, GRb-38, GRb-42, GRb-55, and GRb-59) were grouped into the strongly pathogenic category, with root rot incidence of 51- 70%. Only one isolate (GRb-52) was found to be aggressively pathogenic as they induced root rot more than 70%. On cultivar K-6, all the isolates showed similar pathogenic reactions as with cultivar JL-24 except for four isolates (GRb-20, GRb-24, GRb-28, and GRb-44) that showed minor variation in the degree of pathogenic variability.

**Table 3:** Pathogenic variability of *M. phaseolina* isolates of groundnut collected from Southern India.

Category	Disease Incidence (%)	*M. phaseolina* isolates in different categories	
		Cultivar - JL 24	Cultivar - K-6
Weakly pathogenic	<20	GRb-01, 08, 10, 12, 14, 15, 18, 22, 30, 32, 37, 40, 49, 54, 56	GRb-01, 08, 10, 12, 14, 15, 18, 22, 23, 28, 30, 32, 37, 40, 44, 49, 54, 56
Moderately pathogenic	21-50	GRb-02, 03, 04, 05,06, 07, 09, 13, 17, 19, 21, 23, 24, 25, 26, 28, 29, 31, 33, 34, 35, 36, 39, 41, 43, 44, 45, 46, 47, 48, 50, 51, 53, 57, 58, 60	GRb-02, 03, 04, 05, 06, 07, 09, 13, 17, 19, 20, 21, 24, 25, 26, 29, 31, 33, 34, 35, 36, 39, 41, 43, 45, 46, 47, 48, 50, 51, 53, 57, 58, 60
Strongly pathogenic	51-70	GRb-11, 16, 20, 27, 38, 42, 55, 59	GRb-11, 16, 27, 38, 42, 55, 59
Aggressively pathogenic	>70	GRb-52	GRb-52

### Morphological and cultural characteristics of *M. phaseolina* (groundnut dry root rot) isolates collected from Southern India

A total of 60 isolates of *M. phaseolina* isolated from groundnut plants collected from different states of India were variable in their morphological and cultural characteristics on the PDA medium, as shown in **Supplementary Table 1**.

The time taken to cover the full plate showed a significant variation from 60 to 144 h. Mostly, all the isolates grew very fast and covered the plate within 96 h except for five isolates that grew slowly and showed suppressed growth: GRb05 (120 hrs), GRb09 (132 hrs), GRb04, GRb17, and GRb43 (144 hrs). The appressed growth pattern was observed in 20 isolates, a fluffy growth pattern was observed in 23 isolates, and 17 isolates showed a velvety growth pattern. Colony color *M. phaseolina* isolates varied from light grey to grey and black. Grey colony color was noticed in 36 isolates, light grey in 19 isolates, and black colony color in 5 isolates. Aerial mycelium was seen in most isolates but absent in some isolates (GRb04, GRb05, GRb10, GRb26, GRb30, GRb31, GRb35, GRb39, GRb43, GRb50, GRb58, and GRb60).

The time taken to sclerotia initiation varied from 36 to 72 h after inoculation on Petri plates. In most isolates, sclerotia initiation was observed within 48 h except for isolates GRb04, GRb05, GRb09, GRb17, GRb43, and GRb56, which took up to 72 h. The sclerotial number varied from 14.9 to 49.5 sclerotia per 10X microscopic field and samples were grouped based on the number of sclerotia per 10X microscopic field, *i.e*., less (< 25) in 12 isolates, moderate (25 to 40) in 40 isolates, and high (> 40) in 8 isolates. Based on the diameter of sclerotia size (µm), isolates were grouped into three categories, *i.e*., small size (<90 µm) in 13 isolates, medium size (90 to 120 µm) in 30 isolates, and large (>120 µm) in 17 isolates. The shape of the sclerotia varied among isolates from round (45%), ovoid (33.3%), and irregular (21.6%).

**Figure 4:**
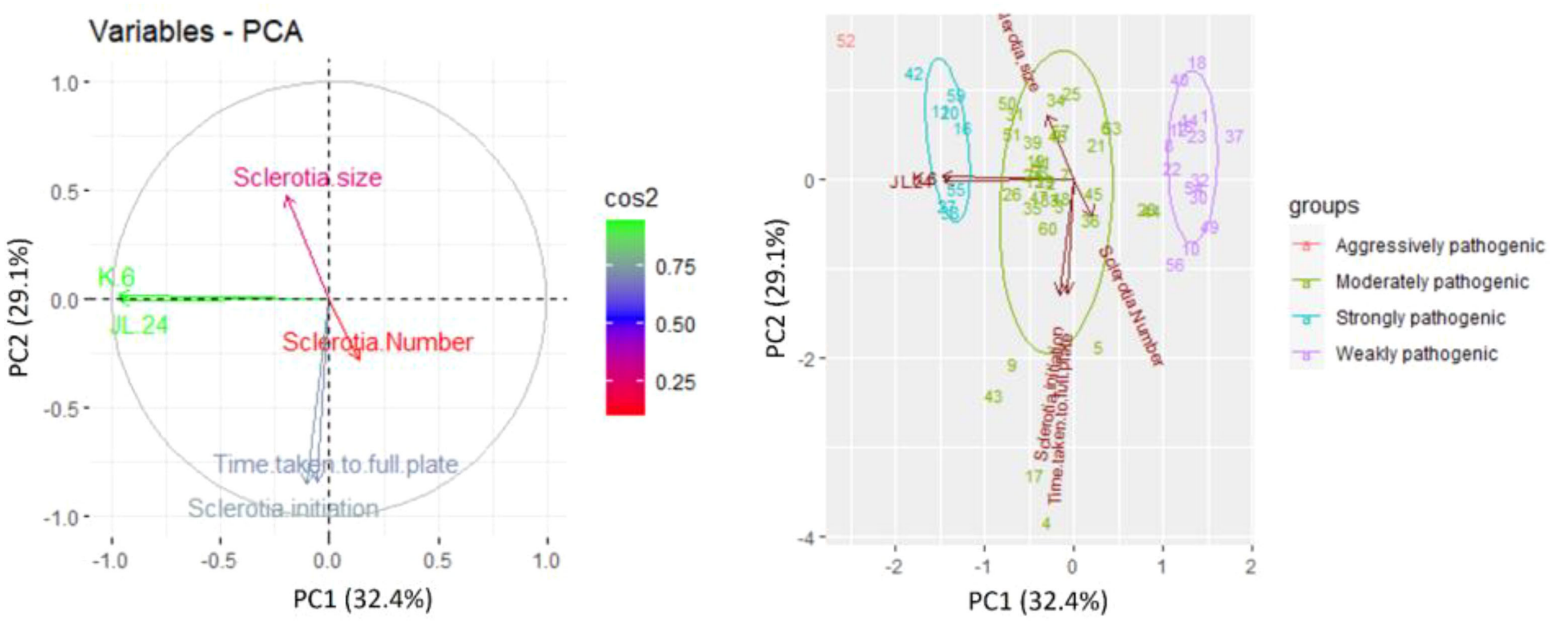
Principal component analysis of *M. phaseolina* isolates based on pathogenic (percent disease incidence on cultivars JL24 and K6) and morphological characteristics (growth rate, sclerotial initiation time, sclerotial number, and sclerotia size). Presented numbers = isolate code.

Morphological and pathogenic variables were subjected to principal component analysis and illustrated by a biplot (**Figure 4**) using R software. The first two principal component axes of the biplot accounted for 32.42% (PC1) and 29.07% (PC2), amounting to a total of 61.49% of total variance. The components showed positive correction with each other except for sclerotial initiation and time taken to cover the full plate (90 mm). All the isolates were grouped based on pathogenic variability (weakly pathogenic, moderately pathogenic, strongly pathogenic, and aggressively pathogenic), among all the isolates, aggressive pathogenic isolate 52 (GRb 52) was located very far from the origin of the biplot.

### Molecular variability of *M. phaseolina* (groundnut dry root rot) isolates collected from Southern India

**Figure 5:**
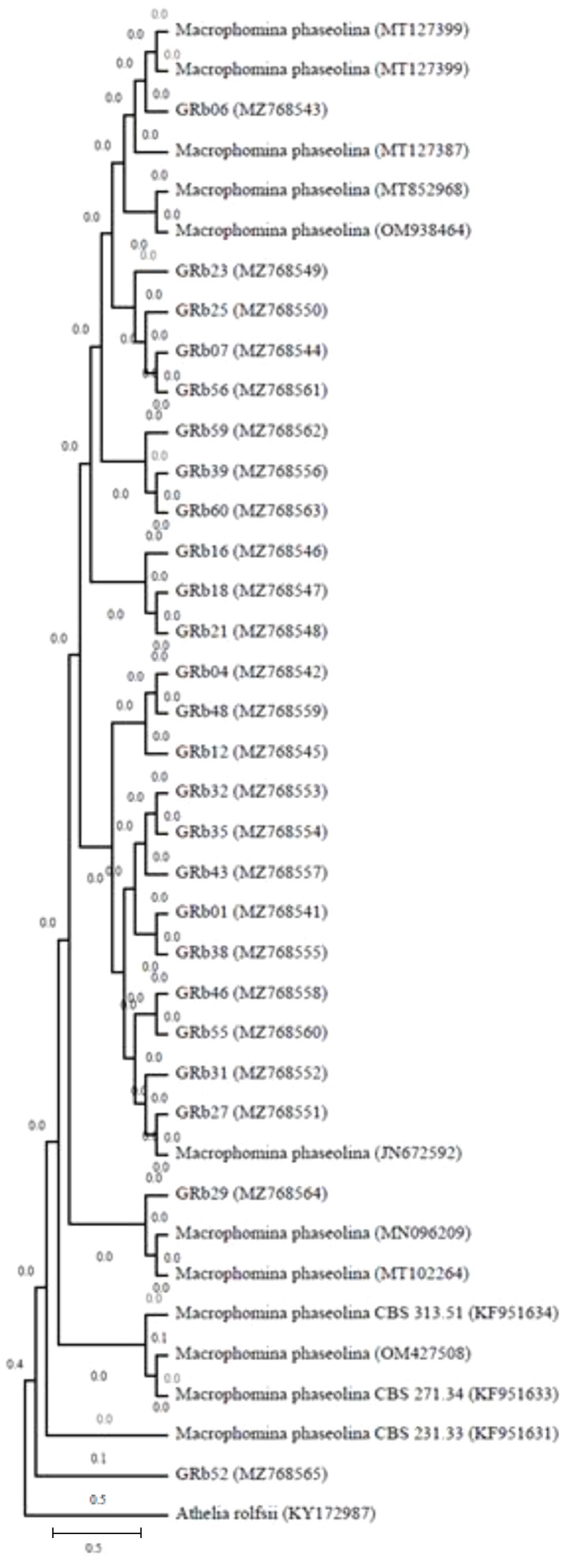
Phylogeny tree showing the relationships among the *M. phaseolina* isolates based on their ITS rDNA sequences. The similarity was analyzed by grouping the neighbor-joining method with 1,000 bootstrapping repetitions to construct the phylogenetic tree (Tamura et al., 2021), and *Athelia rolfsii* was used to root the phylogenetic tree.

Molecular characterization of all the 60 *M. phaseolina* isolates showed amplified rDNA-ITS region fragment length of approximately 600 bp in Gel image (**Supplementary Figure 1**) using ITS1 and ITS4 primers. The 25 rDNA sequences were deposited in the GenBank (NCBI) database under the accession numbers MZ768541– MZ768565. The size of the PCR amplicons ranged from 608 to 622 bp. A phylogenetic tree was constructed for PCR amplicons of the ITS rDNA sequences of the *M. phaseolina isolates* by genetic similarity comparison with data acquired from GenBank (NCBI). The similarity was analyzed by the grouping neighbor-joining method, with 1,000 bootstrapping repetitions in MEGA11 (Tamura et al., 2021). *Athelia rolfsii* was used to root the phylogenetic tree (**Figure 5**).

### Simple sequence repeats analysis

Genetic variability was studied using five SSR primers of MB series against 60 *M. phaseolina* isolates belonging to different states of India, showing 90.4% average polymorphism (**Table 1**). Five primers produced 33 bands with the size varying from 0.1 to 1.4 kb. Out of the 33 bands, 29 bands were polymorphic (87.8%), and 4 were monomorphic. The number of bands varied from 4 to 9, with an average of 6.6 bands per primer. The primers MB-11 (**Supplementary Figure 2**) and MB-13 amplified a maximum of 9 bands within sizes of 200 bp to 1400 bp, showing 88.9% and 77.8% polymorphism, respectively, and both produced a unique monomorphic band that was specific to all the *M. phaseolina* isolates. Whereas primers MB-9 and MB-17 amplified four bands within sizes of 200 bp to 1100 bp, and were highly informative. They showed maximum polymorphism (100%). Primer MB-10 showed 85.7% polymorphism and one monomorphic band size varying from 300 bp to 1100 bp. 

**Figure 6:**
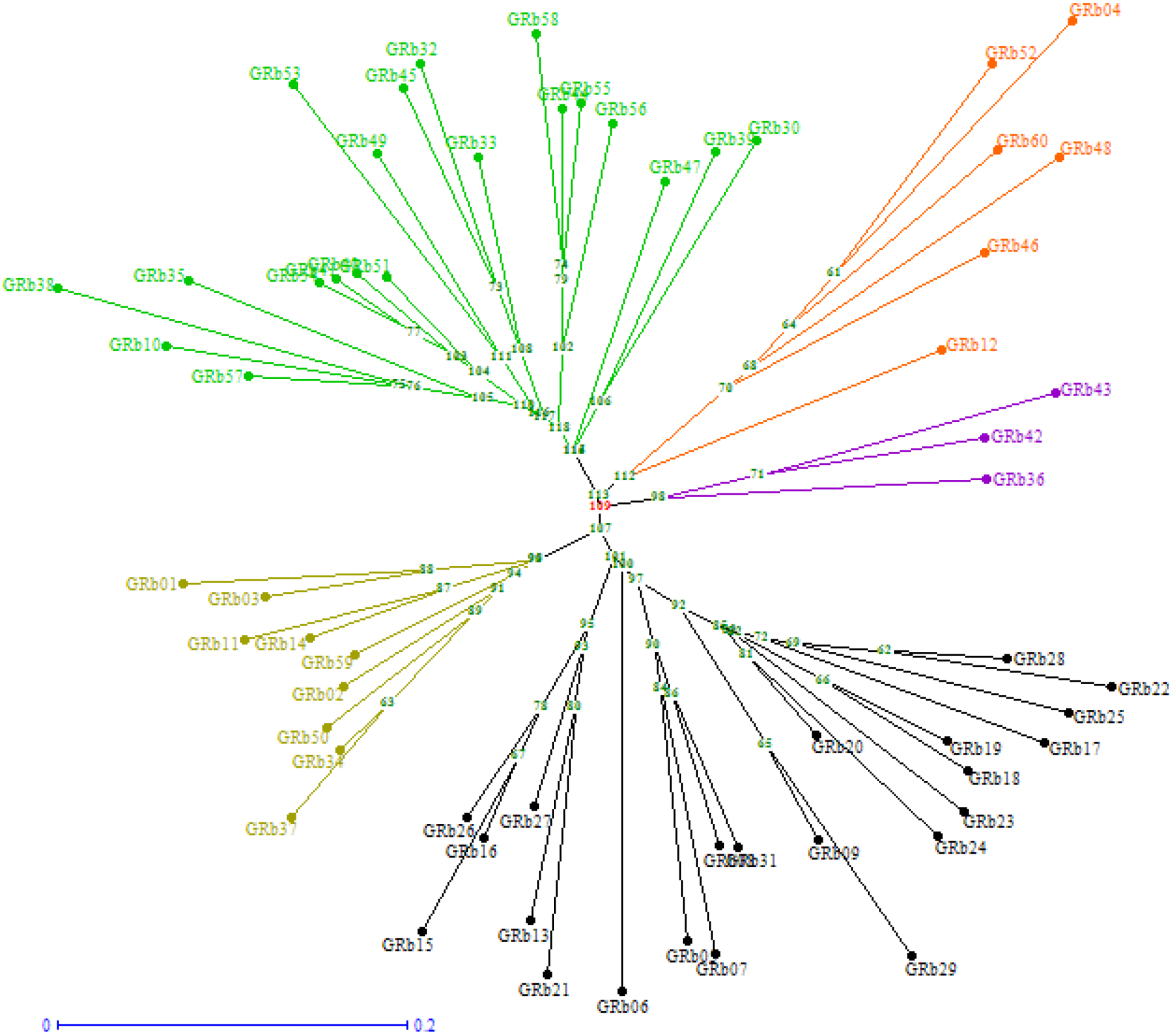
Neighbor-joining tree showing the clustering of 60 *M. phaseolina* isolates (GRb01-GRb60) based on simple sequence repeats (SSR) analysis. The tree was generated using DARWin software (version 6) through unweighted neighbor-joining based on dissimilarity matrices and Jaccard’s coefficient.

The phylogenetic neighbor-joining tree constructed using genotypic data divided 60 isolates into five distinct clusters using the Jaccard coefficient (**Figure 6**). Cluster V was the largest one and accommodated the maximum number of 22 isolates, out of which 13 were from Karnataka, 8 were from Andhra Pradesh, and 1 isolate was from Tamil Nadu. Cluster I was also quite large and accommodated 20 isolates originating from four different states: Telangana (10), Tamil Nadu (8), Andhra Pradesh (1), and Karnataka (1). Cluster II had six isolates, namely, GRb04, GRb12 (Andhra Pradesh), GRb46, GRb48, GRb52, and GRb60 (Telangana). Cluster III had three isolates (GRb36, GRb42, and GRb43) from Tamil Nadu, and Cluster IV had nine isolates from three Indian states: GRb01, GRb02, GRb03, GRb11, and GRb14 from Andhra Pradesh, GRb34 and GRb37 from Tamil Nadu, and GRb50 and GRb59 from Telangana. This result suggests that the molecular variation and differentiation were associated to some extent with geographical origin, but not for all the isolates.

## Discussion

Groundnut dry root rot caused by *M. phaseolina* is an emerging biotic constraint in groundnut growing areas of India due to prolonged moisture stress during the crop growing period. This situation happens primarily because of irregular and scanty rainfall and increasing temperatures. In the present study, a survey was conducted in major groundnut-growing areas of Southern India, and isolates of *M. phaseolina* were collected. The isolates were characterized by morphological, cultural, and molecular characteristics.

Our results on the dry root rot survey in groundnut revealed higher disease incidence in Tamil Nadu and Andhra Pradesh states. A similar percent disease incidence of groundnut dry root rot was reported in the Tiruvannamalai district of Tamil Nadu (Muthukumar et al., 2014) and in the Ananthapurm district of Andhra Pradesh (Veena et al., 2019). The farming situation and soil types also influenced the disease incidence. The groundnut crop grown under irrigated conditions showed less dry root rot incidence than the crop grown under rainfed conditions. The prevalence of dry conditions in rainfed situations might be favorable for the multiplication of pathogens, which could be attributed to the higher level of root rot disease incidence (Muthukumar et al., 2014). The higher disease incidence in rainfed (1.0 to 37.0%) situations and lower disease incidence in irrigated (1.0 to 5.0%) situations were reported in groundnut (Moradia and Khandar, 2011). In the case of soil type, sandy loam had a higher dry root rot incidence than clay loam soil. Rajamohan and Balabaskar (2012) mentioned that the *M. phaseolina* pathogen caused higher root rot disease incidence in sandy soil, whereas incidence was lower in clay soil. In sandy soils, higher disease incidence might be due to the more competitive saprophytic ability of the pathogen at low moisture-holding capacity associated with light soils compared to heavy soils like clay (Umamaheshwari, 1991). Taya et al. (1988) reported that cowpea root rot disease caused by *M. phaseolina* was lower in clay soil, while it was higher in sandy soil.

The relationship between disease incidence and weather parameters showed the higher incidence of disease observed in increased temperature and lower rainfall areas. Pande and Sharma (2014) reported a higher risk of dry root rot when the temperature increases more than 30°C and plotted the correlation coefficient between dry root rot incidence with mean temperature (+0.5) and rainfall (-0.4).

The pathogenic variability showed significant variations among 60 isolates that were grouped into weakly pathogenic (15), moderately pathogenic (36), strongly pathogenic (8), and aggressively pathogenic (1) groups. The pathogenic variability among the isolates of *M. phaseolina* was reported by several studies with different crops. The occurrence of 40 *M. phaseolina* isolates from major soybean-growing states of India was reported (Gade et al., 2018), as well as in safflower (Prasad et al., 2011). The variation in the incidence of root rot disease might be due to the differences in pathogen isolates’ virulence in surveyed locations.

The morphological and cultural studies revealed that *M. phaseolina* isolates were grouped into different categories based on growth pattern (appressed, fluffy, and velvety), growth rate, colony color (light grey, grey, and black), aerial mycelium (present and absent), sclerotial initiation time, sclerotial number (less, moderate, and high), sclerotia size (small, medium, and large) and sclerotia shape (round, ovoid, and irregular). Similarly, earlier studies reported that the *M. phaseolina* associated with different crops also grouped the isolates into different categories based on diameter growth on medium, colony color, mycelial characteristics, the morphology of the sclerotia, and sclerotial initiation time (Suriachandraselvan and Seetharaman, 2003; Sharma et al., 2004; Gupta et al., 2012; Sharma et al., 2012; Tanaji et al., 2017; Wagan et al., 2018; Thirunarayanan et al., 2020; Chiranjeevi et al., 2021).

Molecular studies of the ITS portion of rDNA of all the isolates isolated from different regions of southern India were identical. All the sequences showed 99% similarity with the ITS sequences of *M. phaseolina* isolates in the BLAST search. Several previous studies also used ITS sequencing of rDNA regions to identify *M*. *phaseolina* from different hosts such as chickpeas (Aghakhani and Dubey, 2009; Sharma et al., 2012), legumes (Gautam et al., 2013; Pandey et al., 2021), pigeon pea (Smitha et al., 2016), and safflower (Prasad et al., 2011).

In the molecular studies, five SSR primers of MB series used for 60 *M. phaseolina* isolates belonging to groundnut-growing states of India showed good polymorphism (96.6%). The primers MB 9 and MB 17 showed 100% polymorphism, confirming that these markers are highly suitable for genetic diversity studies in *M. phaseolina*. Jana et al. (2005) reported that SSR markers were highly informative in developing DNA fingerprinting patterns in *Macrophomina phaseolina*. Similarly, Walunj et al. (2018) reported that an average level of polymorphism was 77.96% by eight SSR primers of the MB series, and primer MB-17 showed 100% polymorphic bands. The SSR markers included in the present study proved highly suitable for molecular variability studies in *M. phaseolina*. The existence of molecular diversity among the *M. phaseolina* isolates from the same and different states might be due to mutation in the pathogen in the field, parasexuality, wide host range, and transport of infected seeds, soils, and planting materials (Aghakhani and Dubey, 2009).

The presence of variations among the isolates of *M. phaseolina* in Southern India may be attributed to variations in temperature, soil moisture, soil types, and other edaphic factors and cropping patterns in different locations (Sharma et al., 2012). The pathogenic variability in the *M. phaseolina* isolates may be one of the problems of lack of resistance in the present commercial cultivars of groundnut. The variations in the aggressiveness of *M. phaseolina* isolates cause various degrees of disease incidence in field conditions, may cause yield reduction, and affect groundnut production and productivity in southern India. Pathogenic, morphological, cultural, and molecular variability is imperative for the pathogen to adapt better to diversified environmental behavior and also increases host plant resistance, which leads to the development of varieties resistant to disease and the implementation of new disease management strategies.

## Conclusion

In the present study, evidence was provided on the groundnut dry root rot disease incidence in southern India. It is an emerging disease in groundnuts due to present climatic conditions, and the maximum disease incidence in higher temperature and low rainfall situations was recorded. To the best of our knowledge, this is the first study that provides a detailed description of the pathogenic, morphological, cultural, and molecular diversity existing in the *M. phaseolina* isolates that are affecting groundnut crops in several crop-growing areas of India. This information is extremely useful for pathologists and breeders to identify advanced breeding lines with durable resistance and suitable lines for different groundnut growing areas. Furthermore, the results observed are also helpful in developing disease forecasting models to predict disease incidence. 

## Data availability statement

The original contributions presented in the study are included in the article/**Supplementary Material**, further inquiries can be directed to the corresponding author/s.

## Author contributions

RS and HS conceived and designed the experiments. PP performed the experiments and analyzed the data. PP and HS wrote the paper. KV, RV, and GM gave valuable suggestions to conduct experiments. All authors contributed to the article and approved the submitted version.
