# Supplementary material for: Prevalence of groundnut dry root rot (Macrophomina phaseolina (Tassi) Goid.) and its pathogenic variability in Southern India
Source: Front Fungal Biol. 2023 Dec 4;4:1189043. doi: 10.3389/ffunb.2023.1189043 (PMC10725946; doi:10.3389/ffunb.2023.1189043)
Supplement: Supplementary file 1 [file DataSheet_1.docx]

**Supplementary Tables**

**Supplementary Table 1.** Morphological and cultural characteristics of *M. phaseolina* (groundnut dry root rot) isolates collected from Southern India

| **Isolates** | **Time taken to cover full plate (hrs)*** | **Growth pattern** | **Culture Colour** | **Aerial mycelium** | **Sclerotia initiation (hrs)*** | **Sclerotia Number (No./ 10x microscopic field) **** | **Sclerotia size (µm) ***** | **Sclerotia shape** |
| --- | --- | --- | --- | --- | --- | --- | --- | --- |
|  |  |  |  |  |  |  |  |  |
| GRb01 | 72 | Velvety | Light grey | Present | 36 | 31.2 | 100.7 | Ovoid |
| GRb02 | 72 | Appressed | Grey | Present | 48 | 27.5 | 90.0 | Round |
| GRb03 | 84 | Velvety | Grey | Present | 48 | 27.0 | 93.2 | Irregular |
| GRb04 | 144 | Appressed | Black | Absent | 72 | 43.0 | 72.5 | Round |
| GRb05 | 108 | Appressed | Grey | Absent | 60 | 31.9 | 81.5 | Irregular |
| GRb06 | 72 | Fluffy | Grey | Present | 36 | 28.9 | 86.6 | Ovoid |
| GRb07 | 72 | Fluffy | Grey | Present | 48 | 26.6 | 94.6 | Ovoid |
| GRb08 | 72 | Fluffy | Light grey | Present | 48 | 21.4 | 112.3 | Ovoid |
| GRb09 | 132 | Appressed | Black | Present | 60 | 28.3 | 108.0 | Ovoid |
| GRb10 | 96 | Velvety | Grey | Absent | 48 | 32.1 | 89.2 | Round |
| GRb11 | 72 | Fluffy | Light grey | Present | 36 | 29.7 | 101.7 | Ovoid |
| GRb12 | 84 | Velvety | Light grey | Present | 36 | 29.0 | 108.6 | Round |
| GRb13 | 72 | Appressed | Light grey | Present | 48 | 27.7 | 91.2 | Ovoid |
| GRb14 | 84 | Velvety | Grey | Present | 36 | 27.8 | 115.3 | Ovoid |
| GRb15 | 84 | Appressed | Grey | Present | 36 | 29.3 | 112.5 | Ovoid |
| GRb16 | 72 | Fluffy | Grey | Present | 36 | 31.6 | 90.0 | Irregular |
| GRb17 | 144 | Appressed | Black | Present | 60 | 45.5 | 64.2 | Round |
| GRb18 | 72 | Velvety | Light grey | Present | 36 | 20.5 | 129.9 | Irregular |
| GRb19 | 84 | Velvety | Light grey | Present | 48 | 17.7 | 117.7 | Ovoid |
| GRb20 | 72 | Fluffy | Grey | Present | 48 | 14.9 | 129.9 | Round |
| GRb21 | 84 | Appressed | Grey | Present | 36 | 30.3 | 98.1 | Round |
| GRb22 | 84 | Fluffy | Grey | Present | 48 | 25.3 | 122.6 | Round |
| GRb23 | 60 | Fluffy | Grey | Present | 48 | 31.1 | 112.2 | Irregular |
| GRb24 | 72 | Fluffy | Grey | Present | 48 | 28.6 | 98.8 | Irregular |
| GRb25 | 84 | Velvety | Black | Present | 36 | 20.9 | 127.6 | Ovoid |
| GRb26 | 84 | Appressed | Light grey | Absent | 48 | 41.5 | 126.0 | Round |
| GRb27 | 72 | Fluffy | Light grey | Present | 48 | 49.5 | 102.6 | Round |
| GRb28 | 72 | Fluffy | Grey | Present | 48 | 39.2 | 86.3 | Ovoid |
| GRb29 | 84 | Velvety | Grey | Present | 48 | 27.8 | 115.5 | Ovoid |
| GRb30 | 72 | Velvety | Light grey | Absent | 48 | 37.3 | 93.4 | Round |
| GRb31 | 72 | Appressed | Grey | Absent | 48 | 16.5 | 131.1 | Round |
| GRb32 | 84 | Fluffy | Grey | Present | 36 | 43.6 | 89.1 | Round |
| GRb33 | 72 | Fluffy | Grey | Present | 48 | 38.8 | 92.6 | Irregular |
| GRb34 | 72 | Velvety | Grey | Present | 48 | 18.9 | 147.9 | Round |
| GRb35 | 96 | Appressed | Grey | Absent | 48 | 25.4 | 112.5 | Round |
| GRb36 | 84 | Appressed | Grey | Present | 48 | 36.5 | 95.8 | Round |
| GRb37 | 72 | Fluffy | Grey | Present | 36 | 46.4 | 106.4 | Irregular |

**Supplementary Table 1 (Cont.).** Morphological and cultural characteristics of *M. phaseolina* (groundnut dry root rot) isolates collected from Southern India

| **Isolates** | **Time taken to cover full plate (hrs)*** | **Growth pattern** | **Culture Colour** | **Aerial mycelium** | **Sclerotia initiation (hrs)*** | **Sclerotia Number (No./ 10x microscopic field) **** | **Sclerotia size (µm) ***** | **Sclerotia shape** |
| --- | --- | --- | --- | --- | --- | --- | --- | --- |
|  |  |  |  |  |  |  |  |  |
| GRb38 | 72 | Fluffy | Grey | Present | 48 | 45.1 | 90.5 | Ovoid |
| GRb39 | 96 | Appressed | Light grey | Absent | 36 | 31.2 | 125.2 | Irregular |
| GRb40 | 72 | Appressed | Light grey | Present | 36 | 29.3 | 128.6 | Ovoid |
| GRb41 | 72 | Fluffy | Grey | Present | 48 | 39.3 | 124.3 | Ovoid |
| GRb42 | 72 | Fluffy | Light grey | Present | 36 | 32.4 | 137.5 | Round |
| GRb43 | 144 | Appressed | Black | Absent | 72 | 17.8 | 142.3 | Irregular |
| GRb44 | 72 | Velvety | Light grey | Present | 48 | 44.3 | 92.2 | Round |
| GRb45 | 72 | Velvety | Grey | Present | 48 | 37.5 | 96.8 | Round |
| GRb46 | 72 | Fluffy | Light grey | Present | 36 | 39.2 | 95.4 | Round |
| GRb47 | 72 | Fluffy | Grey | Present | 48 | 26.9 | 76.4 | Ovoid |
| GRb48 | 84 | Fluffy | Grey | Present | 48 | 24.9 | 96.4 | Irregular |
| GRb49 | 72 | Velvety | Light grey | Present | 48 | 38.6 | 69.7 | Round |
| GRb50 | 84 | Appressed | Grey | Absent | 48 | 25.0 | 151.9 | Round |
| GRb51 | 72 | Appressed | Grey | Present | 48 | 35.0 | 141.5 | Irregular |
| GRb52 | 60 | Velvety | Grey | Present | 36 | 28.8 | 136.4 | Ovoid |
| GRb53 | 72 | Fluffy | Grey | Present | 36 | 32.0 | 90.6 | Round |
| GRb54 | 72 | Velvety | Grey | Present | 48 | 39.6 | 104.8 | Ovoid |
| GRb55 | 72 | Fluffy | Grey | Present | 48 | 24.5 | 79.8 | Round |
| GRb56 | 96 | Velvety | Light grey | Present | 60 | 30.8 | 126.5 | Irregular |
| GRb57 | 72 | Fluffy | Grey | Present | 36 | 39.9 | 99.7 | Round |
| GRb58 | 84 | Appressed | Grey | Absent | 48 | 35.7 | 135.0 | Ovoid |
| GRb59 | 72 | Appressed | Light grey | Present | 36 | 32.7 | 119.0 | Round |
| GRb60 | 96 | Appressed | Light grey | Absent | 48 | 21.2 | 89.0 | Round |

* mean of three replications ** mean of ten microscopic fields (10x) *** mean of ten sclerotia

**Supplementary Table 2.** Cultural variability of *M. phaseolina* (groundnut dry root rot) collected from Southern India and assessed on different culture media

| **Isolate** | **PDA** | | | **CDA** | | | **OMA** | | | **RA** | | |
| --- | --- | --- | --- | --- | --- | --- | --- | --- | --- | --- | --- | --- |
|  | **Growth rate (cm) at 72 hrs*** | **Sclerotia initiation (hrs)** | **Sclerotial Production**  ****** | **Growth rate (cm) at 72 hrs** | **Sclerotia initiation (hrs)** | **Sclerotial Production** | **Growth rate (cm) at 72 hrs** | **Sclerotia initiation (hrs)** | **Sclerotial Production** | **Growth rate (cm) at 72 hrs** | **Sclerotia initiation (hrs)** | **Sclerotial Production** |
| GRb01 | 9.0 | 48 | ++++ | 8.1 | 60 | +++ | 9.0 | 48 | ++++ | 7.4 | 72 | +++ |
| GRb02 | 9.0 | 48 | ++++ | 9.0 | 48 | ++++ | 9.0 | 72 | +++ | 7.9 | 72 | +++ |
| GRb03 | 9.0 | 60 | +++ | 7.9 | 60 | ++++ | 9.0 | 60 | +++ | 7.4 | 72 | +++ |
| GRb04 | 6.6 | 84 | ++++ | 4.3 | 108 | ++ | 5.7 | 96 | ++ | 4.6 | 120 | + |
| GRb05 | 6.5 | 72 | ++++ | 4.0 | 96 | ++ | 4.9 | 108 | ++ | 4.9 | 108 | + |
| GRb06 | 9.0 | 48 | ++++ | 9.0 | 48 | ++++ | 8.2 | 72 | +++ | 6.8 | 96 | ++ |
| GRb07 | 9.0 | 48 | ++++ | 9.0 | 48 | ++++ | 7.9 | 84 | +++ | 6.9 | 96 | ++ |
| GRb08 | 9.0 | 48 | ++++ | 9.0 | 48 | ++++ | 9.0 | 72 | +++ | 8.0 | 60 | ++++ |
| GRb09 | 5.7 | 72 | +++ | 2.1 | 108 | ++ | 4.7 | 120 | + | 2.9 | 120 | + |
| GRb10 | 9.0 | 60 | ++++ | 7.8 | 60 | ++++ | 9.0 | 60 | ++++ | 6.8 | 84 | ++++ |
| GRb11 | 9.0 | 48 | ++++ | 9.0 | 60 | ++++ | 9.0 | 60 | ++++ | 7.9 | 72 | +++ |
| GRb12 | 7.9 | 60 | +++ | 7.8 | 60 | +++ | 9.0 | 60 | ++++ | 6.9 | 84 | ++ |
| GRb13 | 7.3 | 48 | ++++ | 6.1 | 60 | ++++ | 4.7 | 84 | +++ | 6.3 | 96 | ++ |
| GRb14 | 9.0 | 48 | ++++ | 7.1 | 96 | ++++ | 9.0 | 48 | ++++ | 9.0 | 48 | ++++ |
| GRb15 | 9.0 | 48 | ++++ | 8.2 | 60 | ++++ | 9.0 | 72 | +++ | 7.0 | 72 | +++ |
| GRb16 | 7.7 | 60 | +++ | 5.9 | 96 | ++ | 7.5 | 84 | +++ | 3.7 | 120 | + |
| GRb17 | 7.4 | 72 | +++ | 5.9 | 108 | ++ | 5.7 | 96 | ++ | 5.0 | 108 | ++ |
| GRb18 | 9.0 | 36 | ++++ | 9.0 | 48 | ++++ | 9.0 | 60 | ++++ | 7.6 | 72 | +++ |
| GRb19 | 9.0 | 48 | ++++ | 9.0 | 48 | ++++ | 8.4 | 72 | +++ | 7.9 | 72 | ++ |
| GRb20 | 9.0 | 48 | ++++ | 9.0 | 48 | ++++ | 9.0 | 60 | ++++ | 8.0 | 72 | +++ |
| GRb21 | 8.0 | 48 | ++++ | 7.4 | 72 | +++ | 9.0 | 72 | +++ | 6.6 | 96 | +++ |
| GRb22 | 9.0 | 48 | ++++ | 9.0 | 60 | ++++ | 8.3 | 84 | +++ | 7.2 | 84 | +++ |
| GRb23 | 4.9 | 72 | +++ | 2.9 | 108 | ++ | 5.5 | 108 | ++ | 3.7 | 120 | +++ |
| GRb24 | 9.0 | 36 | ++++ | 9.0 | 48 | ++++ | 9.0 | 60 | +++ | 8.0 | 60 | ++++ |
| GRb25 | 9.0 | 48 | ++++ | 9.0 | 60 | ++++ | 8.0 | 84 | +++ | 7.8 | 72 | +++ |
| GRb26 | 9.0 | 48 | ++++ | 8.3 | 60 | ++++ | 6.9 | 84 | +++ | 7.5 | 72 | +++ |
| GRb27 | 9.0 | 60 | ++++ | 7.3 | 84 | +++ | 9.0 | 60 | ++++ | 7.1 | 84 | ++ |
| GRb28 | 7.7 | 60 | +++ | 7.4 | 48 | ++++ | 7.4 | 84 | +++ | 7.7 | 72 | ++ |
| GRb29 | 9.0 | 48 | ++++ | 9.0 | 60 | ++++ | 7.4 | 84 | +++ | 6.4 | 96 | + |
| GRb30 | 9.0 | 48 | ++++ | 9.0 | 48 | ++++ | 8.3 | 72 | +++ | 7.4 | 84 | +++ |
| GRb31 | 9.0 | 48 | ++++ | 9.0 | 48 | ++++ | 8.6 | 84 | +++ | 7.3 | 84 | ++ |

**Supplementary Table 2 (Cont.).** Cultural variability of *M. phaseolina* (groundnut dry root rot) collected from Southern India and assessed on different culture media

| **Isolate** | **PDA** | | | **CDA** | | | **OMA** | | | **RA** | | |
| --- | --- | --- | --- | --- | --- | --- | --- | --- | --- | --- | --- | --- |
|  | **Growth rate (cm) at 72 hrs*** | **Sclerotia initiation (hrs)** | **Sclerotial Production**  ****** | **Growth rate (cm) at 72 hrs** | **Sclerotia initiation (hrs)** | **Sclerotial Production** | **Growth rate (cm) at 72 hrs** | **Sclerotia initiation (hrs)** | **Sclerotial Production** | **Growth rate (cm) at 72 hrs** | **Sclerotia initiation (hrs)** | **Sclerotial Production** |
| GRb32 | 7.7 | 60 | ++++ | 7.5 | 84 | ++++ | 8.0 | 84 | +++ | 6.9 | 96 | +++ |
| GRb33 | 8.2 | 60 | +++ | 7.5 | 96 | ++ | 8.4 | 72 | +++ | 6.0 | 108 | ++ |
| GRb34 | 9.0 | 48 | ++++ | 9.0 | 48 | ++++ | 7.8 | 72 | +++ | 7.7 | 72 | +++ |
| GRb35 | 8.3 | 60 | ++++ | 7.6 | 96 | ++ | 8.1 | 84 | +++ | 5.8 | 108 | ++ |
| GRb36 | 8.3 | 60 | +++ | 8.5 | 60 | ++++ | 8.0 | 72 | +++ | 6.4 | 96 | ++ |
| GRb37 | 9.0 | 48 | ++++ | 9.0 | 60 | ++++ | 8.5 | 72 | +++ | 7.1 | 84 | ++ |
| GRb38 | 9.0 | 60 | ++++ | 7.5 | 84 | ++++ | 8.3 | 72 | +++ | 6.5 | 96 | +++ |
| GRb39 | 9.0 | 60 | ++++ | 9.0 | 60 | ++++ | 9.0 | 60 | ++++ | 7.4 | 84 | +++ |
| GRb40 | 9.0 | 48 | ++++ | 9.0 | 60 | ++++ | 8.3 | 60 | +++ | 8.0 | 72 | +++ |
| GRb41 | 8.0 | 60 | ++++ | 8.5 | 72 | ++++ | 8.1 | 84 | +++ | 7.5 | 84 | +++ |
| GRb42 | 9.0 | 36 | ++++ | 9.0 | 36 | ++++ | 8.6 | 60 | ++++ | 8.1 | 72 | ++ |
| GRb43 | 9.0 | 84 | ++++ | 3.6 | 108 | ++ | 5.4 | 96 | ++ | 5.0 | 108 | + |
| GRb44 | 9.0 | 60 | ++++ | 9.0 | 60 | ++++ | 8.7 | 48 | ++++ | 7.1 | 84 | ++ |
| GRb45 | 9.0 | 60 | ++++ | 8.4 | 60 | ++++ | 8.7 | 72 | +++ | 6.5 | 96 | ++ |
| GRb46 | 4.3 | 72 | +++ | 7.2 | 60 | +++ | 8.3 | 60 | +++ | 4.2 | 108 | ++ |
| GRb47 | 9.0 | 48 | ++++ | 9.0 | 60 | ++++ | 9.0 | 48 | ++++ | 7.6 | 84 | +++ |
| GRb48 | 9.0 | 48 | ++++ | 8.6 | 60 | ++++ | 9.0 | 48 | ++++ | 7.0 | 84 | ++ |
| GRb49 | 9.0 | 48 | ++++ | 8.2 | 60 | ++++ | 9.0 | 60 | +++ | 7.7 | 72 | +++ |
| GRb50 | 7.9 | 60 | ++++ | 7.8 | 60 | ++++ | 9.0 | 72 | +++ | 6.6 | 84 | ++ |
| GRb51 | 9.0 | 48 | +++ | 9.0 | 60 | ++++ | 8.3 | 84 | +++ | 7.5 | 84 | ++ |
| GRb52 | 9.0 | 48 | ++++ | 8.5 | 60 | ++++ | 9.0 | 48 | ++++ | 8.0 | 72 | +++ |
| GRb53 | 9.0 | 60 | ++++ | 7.8 | 72 | +++ | 9.0 | 72 | ++++ | 6.7 | 96 | +++ |
| GRb54 | 9.0 | 60 | +++ | 8.2 | 72 | +++ | 9.0 | 72 | +++ | 6.7 | 96 | ++ |
| GRb55 | 9.0 | 48 | ++++ | 8.1 | 60 | ++++ | 9.0 | 60 | +++ | 7.2 | 84 | +++ |
| GRb56 | 9.0 | 60 | ++++ | 8.2 | 60 | ++++ | 9.0 | 72 | +++ | 6.8 | 96 | ++ |
| GRb57 | 9.0 | 48 | ++++ | 7.8 | 84 | +++ | 9.0 | 60 | ++++ | 7.3 | 84 | +++ |
| GRb58 | 9.0 | 60 | ++++ | 7.6 | 72 | +++ | 9.0 | 60 | +++ | 6.9 | 108 | ++ |
| GRb59 | 9.0 | 36 | ++++ | 9.0 | 36 | ++++ | 9.0 | 72 | +++ | 8.4 | 60 | ++++ |
| GRb60 | 8.4 | 60 | +++ | 7.7 | 72 | +++ | 9.0 | 60 | ++++ | 7.4 | 96 | ++ |
| Mean | 8.5 | 54.6 |  | 7.8 | 68.2 |  | 8.1 | 69.6 |  | 6.8 | 86.8 |  |
| Sem | 0.33 |  |  | 0.41 |  |  | 0.29 |  |  | 0.26 |  |  |
| CD (0.01) | 0.94 |  |  | 1.15 |  |  | 0.82 |  |  | 0.74 |  |  |
| CV% | 0.83 |  |  | 1.02 |  |  | 0.73 |  |  | 0.65 |  |  |

**Note:** + = Poor, ++ = Moderate, +++ = Good, ++++ = Excellent. * mean of three replications ** number sclerotia/ 10X microscopic field

**Supplimentary Figures**


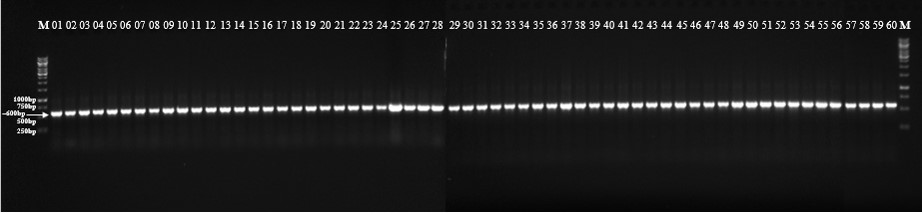


**Supplimentary Figure 1.** Identification of *M. phaseolina* isolates with the universal primers ITS1 and ITS4, Amplified band size of approximately 600 bp. Line 1-60: *R. bataticola* isolates (GRb01-GRb60); M = 1 Kb ladder


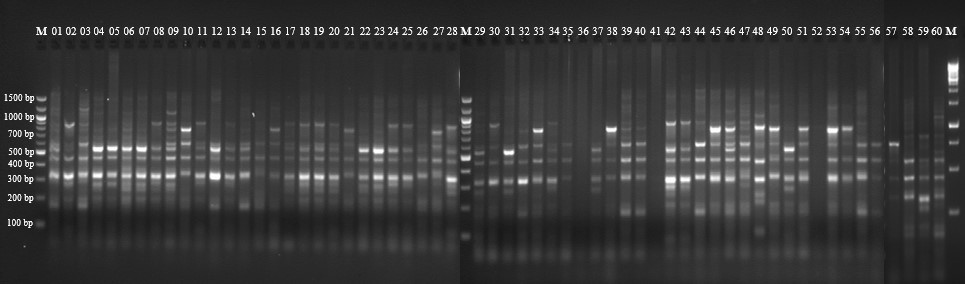


**Supplimentary Figure 2.** DNA profile generated by simple sequence repeat (SSR) primers MB 11; M = 100 bp marker on left and middle and 1 kb marker on right; lanes 1–16 (Andhra Pradesh), 17– 30 (Karnataka), 31–44 (Tamil Nadu) and 45–60 (Telangana) represents the 60 isolates (GRb01-GRb60) of *M. phaseolina.*
